# HDAC8, A Potential Therapeutic Target for the Treatment of Malignant Peripheral Nerve Sheath Tumors (MPNST)

**DOI:** 10.1371/journal.pone.0133302

**Published:** 2015-07-22

**Authors:** Gonzalo Lopez, Kate Lynn J. Bill, Hemant Kumar Bid, Danielle Braggio, Dylan Constantino, Bethany Prudner, Abeba Zewdu, Kara Batte, Dina Lev, Raphael E. Pollock

**Affiliations:** 1 Comprehensive Cancer Center, The Ohio State University, Columbus, OH, United States of America; 2 Surgery B, Sheba Medical Center, Tel Aviv, Israel; 3 Surgical Oncology, The Ohio State University, Columbus, OH, United States of America; German Cancer Research Center, GERMANY

## Abstract

**Introduction:**

HDAC isoform-specific inhibitors may improve the therapeutic window while limiting toxicities. Developing inhibitors against class I isoforms poses difficulties as they share high homology among their catalytic sites; however, HDAC8 is structurally unique compared to other class I isoforms. HDAC8 inhibitors are novel compounds and have affinity for class I HDAC isoforms demonstrating anti-cancer effects; little is known about their activity in malignant peripheral nerve sheath tumors (MPNST). Recently, we demonstrated anti-MPNST efficacy of HDAC8i in human and murine-derived MPNST pre-clinical models; we now seek to consider the potential therapeutic inhibition of HDAC8 in MPNST.

**Methods:**

Four Human MPNST cell lines, a murine-derived MPNST cell line, and two HDAC8 inhibitors (PCI-34051, PCI-48012; Pharmacyclics, Inc. Sunnyvale, CA) were studied. Proliferation was determined using MTS and clonogenic assays. Effects on cell cycle were determined via PI FACS analysis; effects on apoptosis were determined using Annexin V-PI FACS analysis and cleaved caspase 3 expression. *In vivo* growth effects of HDAC8i were evaluated using MPNST xenograft models. 2D gel electrophoresis and mass spectrometry were used to identify potential HDAC8 deacetylation substrates.

**Results:**

HDAC8i induced cell growth inhibition and marked S-phase cell cycle arrest in human and murine-derived MPNST cells. Relative to control, HDAC8i induced apoptosis in both human and murine-derived MPNST cells. HDAC8i exhibited significant effects on MPNST xenograft growth (p=0.001) and tumor weight (p=0.02). Four potential HDAC8 substrate targets were identified using a proteomic approach: PARK7, HMGB1, PGAM1, PRDX6.

**Conclusions:**

MPNST is an aggressive sarcoma that is notoriously therapy-resistant, hence the urgent need for improved anti-MPNST therapies. HDAC8 inhibition may be useful for MPNST by improving efficacy while limiting toxicities as compared to pan-HDACis.

## Introduction

Recently developed HDAC-specific inhibitors have been used to expand knowledge of isoform-specific contributions to cellular function; these include HDAC6 (e.g. tubacin, tubastatin a), HDAC8 (PCI-34051), and HDAC3 (RGFP966). Of note, some of these “isoform-specific” compounds demonstrate varying affinity to HDAC isoforms other than their intended target [1]. Within class I, HDAC8 is structurally distinct [2] versus other isoforms within this class, leading to the development of HDAC8-specific inhibitors. Differentiating characteristics of HDAC8 from other class I isoforms (HDAC1, HDAC2, HDAC3) is the lack of a 50–111 amino acid C-terminal domain which is important for enzyme recruitment, as well as a shorter N-terminal L1 loop by two residues [3]. Compared to other class I isoforms, HDAC8 is not phosphorylated by CK2, but by PKA (cyclic AMP-dependent protein kinase A) [4].

The role of HDAC8 in normal and cancer cells remains unexplored. Hyperacetylation of core histone proteins yields conflicting results: HDAC8 can deacetylate histone 3 and 4 in some, but not all cell types [4], [5]. Potential deacetylation targets of HDAC8 include estrogen-related receptor alpha (ERRα) [6], inv-16 fusion protein [7], and CREB [8]. HDAC8 also functions in non-deacetylation roles. Lee et al. [9] demonstrated phosphorylated-HDAC8 interacts with human ever shorter telomeres 1B (hEST1B) by recruiting Hsp70 to a complex that inhibits C-terminal heat shock protein interacting protein (CHIP) independent of its acetylation state. Cytoplasmic HDAC8 also interacts with smooth muscle alpha-actin (α-SMA) in muscle cells undergoing differentiation in a non-deacetylase capacity [10]. In a potential clinical setting, cytoplasmic HDAC8 has been demonstrated to play a potential diagnostic role in mesenchymal tumors of the uterus [11]. These intriguing observations provide an impetus for developing novel small molecules to target HDAC8; these include compound 2/HDAC inhibitor XIX, PCI-34051, and PCI-48012.

PCI-34051 (PCI3) is a potent HDAC8-specific inhibitor with a 4,200-fold selectivity over other HDAC isoforms. It induces apoptosis in T-cell lymphoma and leukemia cells lines; however, no significant apoptosis was observed in B-cell or solid tumor cell lines. Moreover, PCI3 did not induce the hyper-acetylation of target histones or tubulin in the cell lines tested [12].

In neuroblastoma, HDAC8 expression was prognostic for an unfavorable outcome [13]. Compound 2, a linker-less hydroxamate HDAC8 inhibitor, was tested in neuroblastoma cell lines; siRNA knockdown of HDAC8 as well as inhibition with compound 2 induced differentiation by stimulating neuritic-like structural outgrowth and abrogating cell proliferation without apoptosis induction [14]. HDAC8i also induced increased expression of p21Waf1/Cip1 and NTRK1/TrkA which was associated with cell line growth inhibition [13], [15]. Intriguingly, neuroblastoma and MPNST both arise from neural crest cell origins, suggesting a possible role for HDAC8 in progression of these cancers.

## Materials and Methods

### Cell lines and reagents

Human MPNST cell lines: S462 (provided by Dr. Lan Kluwe, University Hospital Eppendorf, Hamburg, Germany [16]), ST88 (provided by Dr. Jonathan Fletcher, Brigham and Women’s Hospital, Boston, MA [17]), STS26T (provided by Dr. Steven Porcelli, Albert Einstein College of Medicine, Bronx, NY[18]), MPNST724 (provided by Dr. Jonathan Fletcher [19]), MPNST642 (isolated in our laboratory [20], MD Anderson Cancer Center, Houston, TX). Murine-derived MPNST cell line: MPNST6IEPVI. Human MPNST cell lines were previously used in our lab [20], [21]. MPNST6IEPVI was provided by Dr. Luis Parada (UT Southwestern, Dallas, TX) [22]. All MPNST cell lines were cultured in DMEM 1X supplemented with 10% FBS/ 5% Pen Strep (Life Technologies). The HDAC inhibitors PCI2, PCI-34051, and PCI-48012 were obtained from Pharmacyclics, Inc (Sunnyvale, California). Commercial antibodies used for Western blot analysis: acetylated Histone 3 (EMD Millipore, Cat# 06–599, Rabbit polyclonal), acetylated Histone 4 (EMD Millipore, Cat# 06–866, Rabbit polyclonal); acetylated tubulin (Sigma, Cat# T7451, Mouse monoclonal); cleaved caspase 3 (Cell Signaling, Cat# 9661, Rabbit polyclonal), β-actin (Santa Cruz, Cat# sc-47778, Mouse monoclonal). Secondary antibodies included horseradish peroxidase–conjugated anti-rabbit (Santa Cruz, Cat# sc-2357) and anti-mouse (Santa Cruz, Cat# sc-358920).

### Western Blot analyses

Western blots were performed by standard methods. 50 μg of protein extract from cultured cells was separated using SDS-PAGE then transferred onto PVDF membranes. Membranes were blocked with 5% milk and blotted overnight with respective antibodies. HRP-conjugated secondary antibodies were detected using Western Lightning Plus-ECL (PerkinElmer, Inc).

### Growth studies: MTS, Clonogenicity, Cell cycle, apoptosis Assay

MTS, clonogenicity, cell cycle, and annexin V assays were performed as previously described [20], [23]. Apoptosis was measured using the Apoptosis Detection kit I (BD Biosciences) per manufacturer's recommendations.

### *In vivo* therapeutic experiments

All animal procedures and care were approved by the MD Anderson Cancer Center Institutional Animal Care and Usage Committee. Animals received humane care as per the Animal Welfare Act and the NIH "Guide for the Care and Use of Laboratory Animals". For experiments evaluating effect of PCI-48012 (PCI4) monotherapy on local tumor growth trypan blue staining confirmed viable MPNST6IEPVI cells. Cell suspensions (1 x 10^6^) were injected subcutaneously into the flank of 6–7 week old female hairless SCID mice and growth was measured twice weekly. After establishment of palpable lesions (average diameter ~4–5mm), mice were randomly assigned to receive either vehicle control or PCI4 (20mg/kg BID). PCI4 was solubilized in 5% Methylcellulose and administered i.p. twice daily, five days/week. Treatment continued until mice in control group mandated euthanasia. Euthanasia occurred when tumors reached 1.5mm in diameter. IACUC approved tumor sizes up to 1.5cm in diameter. Tumors were resected, weighed, and stored for further use.

### Proteomics

Two-dimensional electrophoresis was performed according to the carrier ampholine method of isoelectric focusing [24], [25] by Kendrick Labs, Inc. (Madison, WI) as follows: Isoelectric focusing was carried out in a glass tube of inner diameter 2.3 mm using 2% pH 3.5–10 ampholines mix4LServalytes (Serva, Heidelberg, Germany) for 9600 volts-hrs. One ug of an IEF internal standard, tropomyosin, was added to the sample. This protein migrates as a doublet with lower polypeptide spot of MW 33,000 and pI 5.2. The enclosed tube gel pH gradient plot for this set of ampholines was determined with a surface pH electrode.

After equilibration for 10 min Buffer ‘O’ (10% glycerol, 50 mM dithiothreitol, 2.3% SDS and 0.0625 M tris, pH 6.8), each tube gel was sealed to the top of a stacking gel that overlaid a 10% acrylamide slab gel (0.75 mm thick). SDS slab gel electrophoresis was carried out for about 4 hrs at 15 mA/gel. After slab gel electrophoresis, the gels were placed in the transfer buffer (10 mM Caps, pH 11.0, 10% methanol) and transblotted onto a PVDF membrane overnight at 200 mA and approximately 100 volts/two gels. The following proteins (Sigma Chemical Co., St. Louis, MO) were used as molecular weight standards: myosin (220,000), phosphorylase A (94,000), catalase (60,000), actin (43,000), carbonic anhydrase (29,000), and lysozyme (14,000). These standards appear as bands at the basic edge of the Coomassie Brilliant Blue R-250-stained membrane.

Coomassie-stained blots were scanned. The blots were blocked for two hrs in 5% BSA in Tween-20 tris buffered saline (TTBS) and rinsed in TTBS. The blots were then incubated in primary antibody (anti-Acetylated lysine [Cell Signaling, Cat. #9441, Lot #10] diluted 1:1,000 in 2% BSA) overnight and rinsed 3X 10 min in TTBS. The blots were then placed in secondary antibody (anti-rabbit IgG-HRP [GE Healthcare, Cat. #NA934V, Lot #4646554] 1:2,000 diluted in TTBS) for 2 hrs, rinsed in TTBS as above, treated with ECL, and exposed to X-ray film.

Western blot films (15 min exposures) were obtained from the sample and scanned with a laser densitometer (Model PDSI, Molecular Dynamics Inc, Sunnyvale, CA). The scanner was checked for linearity prior to scanning with a calibrated Neutral Density Filter Set (Melles Griot, Irvine, CA). The images were analyzed using Progenesis Same Spots software (version 4.5, 2011, Nonlinear Dynamics, Durham, NC) and Progenesis PG240 software (version 2006, Nonlinear Dynamics, Durham, NC). The general method of computerized analysis for these pairs included image warping in conjunction with detailed manual checking.

Spot % is equal to spot integrated density above background (volume) expressed as a percentage of total density above background of all spots measured. Difference is defined as fold-change of spot percentages.

### Statistical analyses

Cell culture-based assays were repeated at least 3X; mean standard deviation was calculated. Cell lines were examined separately. For outcomes that were measured at a single time point, 2-sample t-tests were used to assess the differences. Differences in xenograft growth *in vivo* were assessed using a 2-tailed Student's t-test. Significance was set at *p<0.05 and **p<0.01.

## Results

### HDAC8 inhibition abrogated human and murine-derived MPNST cell growth *in vitro*

**Fig 1:**
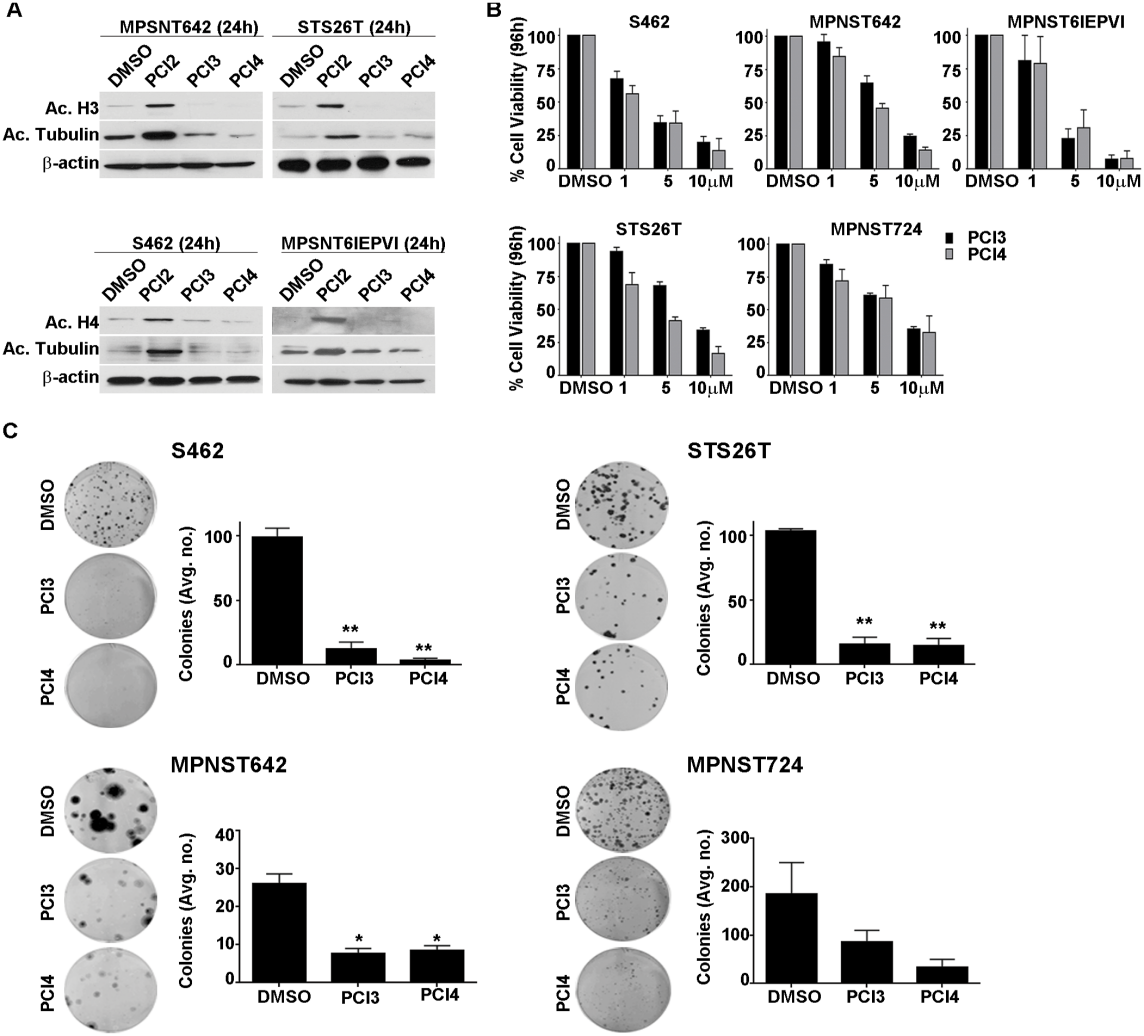
The effect of HDAC8 inhibition on MPNST. **A.** Western blot analysis showing PCI3 and PCI4 (each 5μM/24h) do not induced increases in histone 3, histone 4, and tubulin acetylation in human (STS26T, MPNST642, S462 cells) and murine-derived (MPNST6IEPVI) MPNST cells. PCI2 (0.5μM/24h) was used as a positive control for these experiments. **B.** MTS assays demonstrating the effect of HDAC8 inhibition (96h) on human and murine-derived MPNST cell growth. Murine-derived MPNST cells (MPNST6IEPVI) exhibited marked sensitivity to both HDAC8i compared to human MPNST cell lines. Black bars depict PCI3, grey bars depict PCI4. **C.** HDAC8i abrogates human MPNST cell colony formation capacity. 100–200 cells were initially plated for each cell line. Twenty-four hours after plating, PCI3/4 (5μM) was added to the treatment wells continuously for 10–14 days. Graphs depict the average number of colonies counted in the experiments. Murine-derived MPNST cells failed to grow colonies at 100–500 cells per well, therefore the clonogenic capacity of murine MPNST cells are yet to be determined. * p<0.05, ** p<0.01.

Two HDAC8 inhibitors were used: PCI-34051 (PCI3) and PCI-48012 (PCI4), and a pan-HDACi PCI2 was used as a control. PCI-48012 is a variant compound with greater stability and PK/PD (pharmacokinetics/pharmacodynamics) than PCI-34051 and can be used *in vivo*. The effect of PCI3 (5μM/24h) and PCI4 (5μM/24h) compared to PCI2 (0.5μM/24h) on acetylated target expression was evaluated in human (STS26T, MPNST642, S462) and murine-derived (MPNST6IEPVI) MPNST cell lines (Fig 1A). Time-dependent protein acetylation increases in cells treated with PCI2, but not in cells treated with either HDAC8 inhibitor indicated that tubulin, histone 3, and histone 4 were not substrate targets of HDAC8 in MPNST. While these initial experiments did not reveal acetylation targets of HDAC8, the effect of both compounds on MPNST cell growth were determined using MTS assays. Both human and murine-derived MPNST cell lines were treated with PCI3 and PCI4 for 96h (Fig 1B). HDAC8 inhibition abrogated human and murine-derived MPNST cell line growth. MTS data demonstrated similar effect of both compounds in all cell lines tested. Murine-derived MPNST cell lines appeared to have a greater sensitivity to both compounds as compared to human MPNST cell lines. MPNST cell growth was further evaluated using clonogenic assays (Fig 1C). Murine-derived MPNST cells did not form colonies and so were not used for this experiment. Human MPNST cell lines were plated at 100–200 cells per well. Twenty four hours after plating, cells were treated with PCI3 or PCI4. Cells were continuously treated with compounds every 72h for 10–14 days. Data demonstrated significant HDAC8 inhibition of human MPNST cell line clonogenic potential. Taken together, these data demonstrate the inhibitory impact of pharmacological inhibition of HDAC8 on human and murine-derived MPNST growth.

### HDAC8 inhibition induced S-phase cell cycle arrest and apoptosis in human and murine-derived MPNST cells

**Fig 2:**
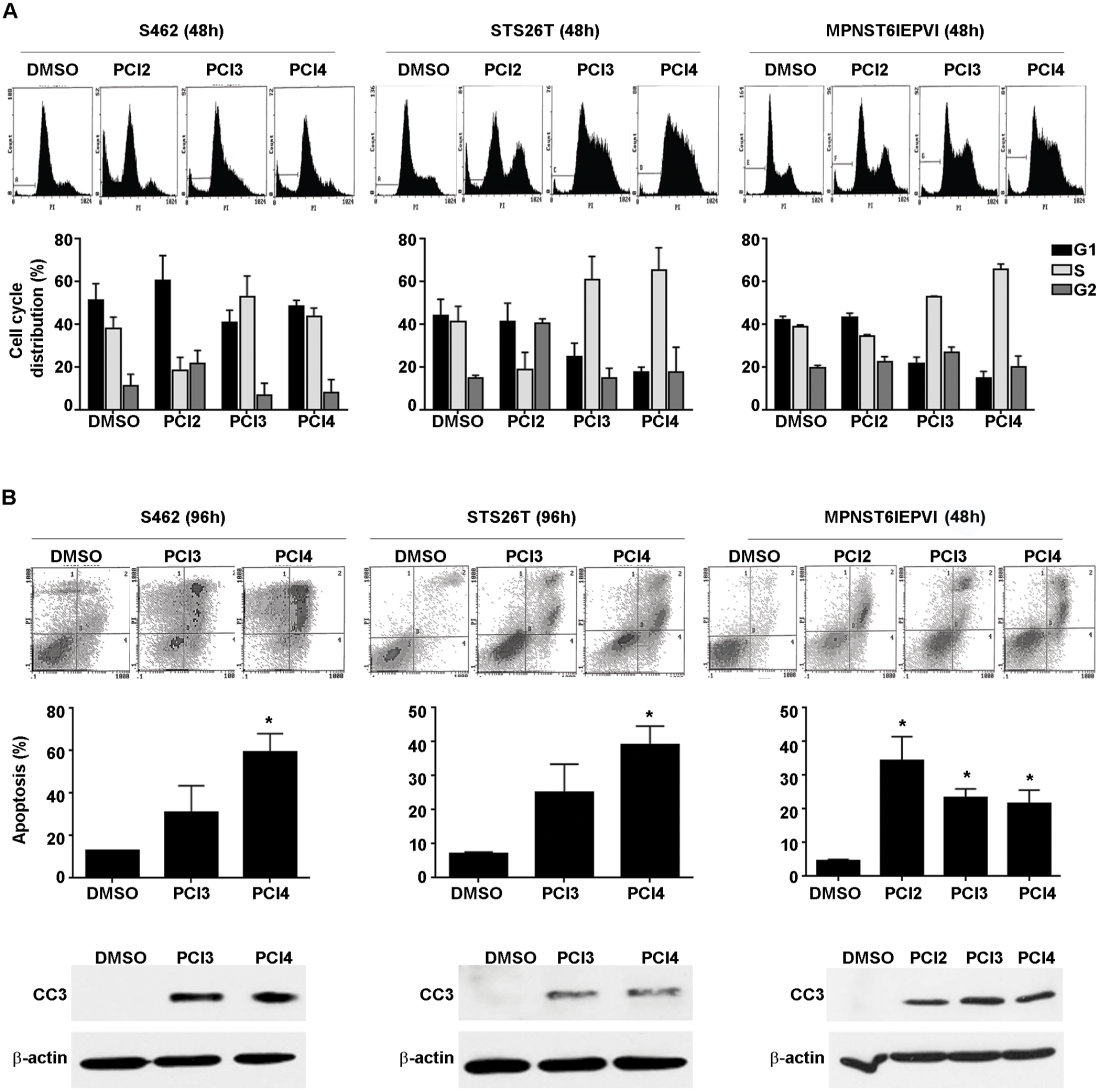
Effects of HDAC8 inhibition on MPNST cell cycle and apoptosis. **A.** PI staining/FACS analyses showing the effect of HDACis (PCI2 0.5μM/48h, PCI3/4 5μM/48h) on human and murine-derived MPNST cell cycle progression. PCI2 reduced S-phase with a modest increase in G1 and G2 in S462 cells. STS26T exhibited a PCI2-induced G2 arrest and a decrease in S-phase. The effect of PCI2 on murine-derived MPNST cell cycle was modest. All 3 MPNST cell lines exhibited S-phase arrest when treated with either HDAC8i. Additionally, an increase in sub G1 population is observed (depicted in the respective histograms), suggesting cell death. **B.** HDAC8i (PCI3/4 5μM/96h) induced human MPNST cell apoptosis (Annexin V/PI staining FACS analyses). The response of these cell lines to HDAC8i-induced apoptosis was recapitulated and further confirmed via WB for cleaved caspase 3 (CC3). Due to the higher sensitivity toward HDAC8i compared to human MPNST cell lines, murine MPNST cells were treated for 48h. PCI2 (0.5μM/48h) and both HDAC8i (PCI3/4 5μM/48h) induced marked apoptosis in murine-derived MPNST cells (Annexin V/PI staining FACS analyses, CC3 WB). * p<0.05.

To potentially identify the underlying contributions of HDAC8 inhibition on MPNST cell growth, PCI3 and PCI4 were evaluated for their effects on cell cycle; PCI2 was used as a control (Fig 2A). Both PCI3 and PCI4 (each 5μM/48h) induced an increase in S-phase in all cell lines tested. PCI2 (0.5μM/48h) decreased S-phase in S462 cells and STS26T with a modest decrease in MPNST6IEPVI. PCI2 increased G1 and G2/M in S462 cells with a marked G2/M-arrest in STS26T. The effects of PCI2 on cell cycle in MPNST6IEPVI were modest vs DMSO control. Despite differences in species of origin, and NF1 status, HDAC8 inhibition-induced S-phase arrest in all cell lines tested.

Cell cycle histograms also revealed an increase in the sub-G1 population after PCI2, PCI3, and PCI4 treatment, suggesting fragmented DNA/apoptosis. To examine the possibility of drug-induced apoptosis, Annexin V/PI FACS analysis and the expression of cleaved caspase 3 (CC3) were used. S462 cells and STS26T were treated with PCI3 and PCI4 for 96h. Due to a greater sensitivity in murine-derived MPNST cells, MPNST6IEPVI was treated for 48h; PCI2 served as a control in MPNST6IEPVI cells. The effect of PCI2 on apoptosis in human MPNST cell lines was previously determined [18]. PCI3 and PCI4 induced a marked increase in Annexin V/PI positive cells and increased CC3 protein expression in all cell lines tested (Fig 2B). PCI4 treatment induced a significant increase in Annexin V/PI positive cells compared to PCI3 in S462 cells and STS26T, whereas PCI2/PCI3/PCI4 significantly impacted apoptosis in MPNST6IEPVI. The amount of HDAC8i-induced apoptosis-positive cells appeared to be higher in S462 cells vs STS26T cells. This data recapitulates previously reported data [18] where NF1-associated cells (S462 cells) exhibited greater sensitivity to HDAC inhibition compared to sporadic cells (STS26T). This dichotomy *vis-á-vis* other HDAC isoforms is yet to be determined.

### HDAC8 inhibition demonstrated a modest effect on murine-derived MPNST xenograft tumor growth *in vivo*

**Fig 3:**
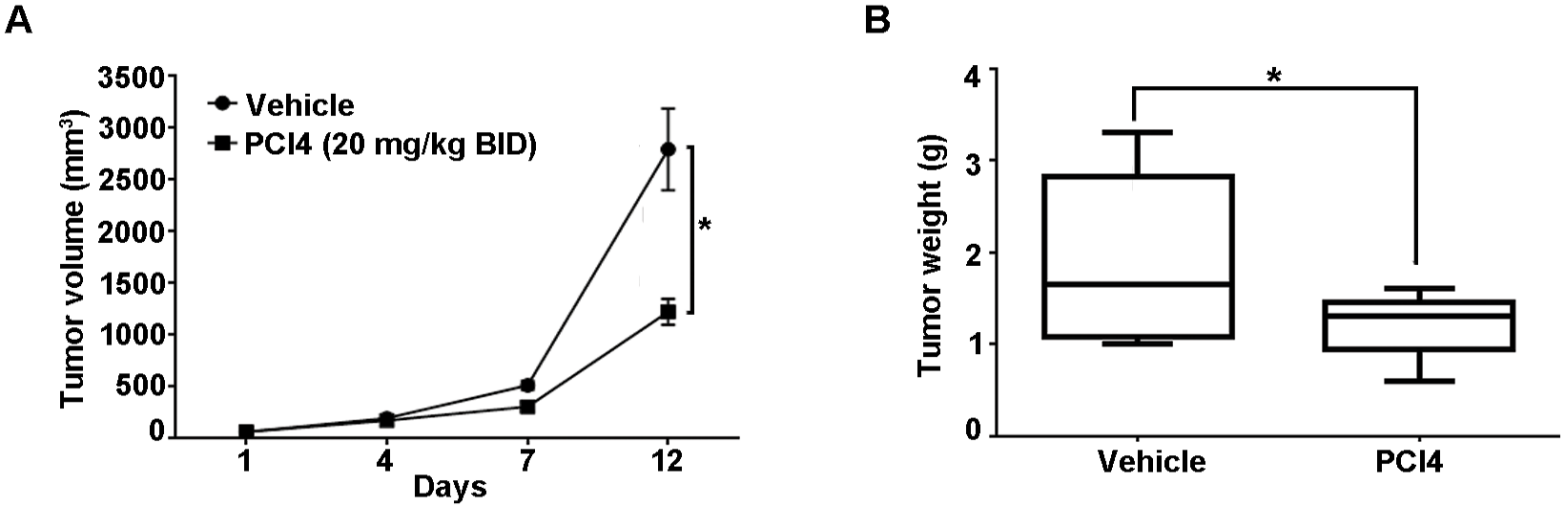
Effect of HDAC8 inhibition *in vivo*. SCID mice bearing MPNST6IEPVI xenografts were treated with PCI4 (20 mg/kg BID) or vehicle (10 mice/group). Tumor growth and weight curves are depicted showing that PCI4 abrogated the growth of MPNST6IEPVI tumors (p = 0.001 and 0.02 for tumor size and weight, respectively).

Next, the effect of HDAC8 inhibition was evaluated *in vivo*. MPNST6IEPVI xenograft model was used and treated with vehicle (control) or PCI4 (20 mg/kg BID). No discernable therapy-induced toxicities were observed in the mice. MPNST6IEPVI tumor growth was rapid; experiment conducted for 12 days. Upon completion, PCI4 induced significant decreases in both tumor volume (p = 0.001) and tumor weight (p = 0.02) compared to vehicle (Fig 3A). This short treatment course demonstrates a potential cytostatic effect of HDAC8 inhibition on MPNST6IEPVI xenografts.

### Potential HDAC8 acetylation targets identified in a human MPNST cell line

**Fig 4:**
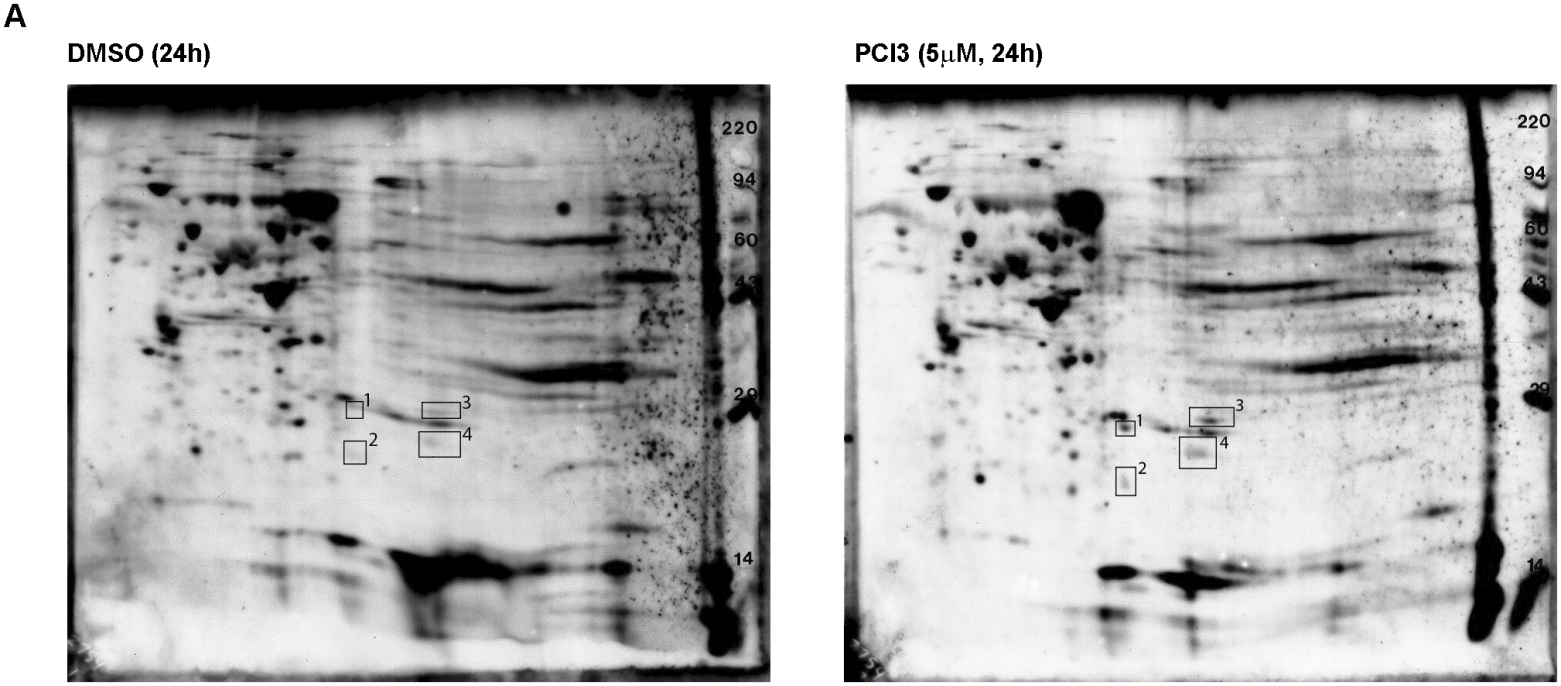
Potential HDAC8 acetylation targets. Proteomics analysis of potential HDAC8 acetylation substrates. Differences in spots (pan-acetyl lysine) in S462 cells treated with DMSO or PCI3 (5μM/24h) were analyzed. Several spots were then analyzed via MS yielding potential HDAC8 acetylation targets: PRDX6, PGAM1, HMGB1, PARK7/DJ-1.

Little is known about acetylation targets of HDAC8. We next aimed to identify potential acetylation targets in an MPNST cell line. S462 cells were treated with PCI3 (5μM/24h) and samples were outsourced (Kendrick Labs, Inc) for proteomics analysis. 2D electrophoresis and mass spectrometry generated four potential HDAC8 acetylation targets in S462 cells: peroxiredoxin 6 (PRDX6), phoshoglycerate mutase 1 (PGAM1), high mobility group protein B1 (HMGB1), and Parkinson protein 7 (PARK7/DJ-1) (Fig 4 and Table 1). These four proteins range from 25–28 kDa (kilodalton), thus resulting in potential technical difficulties with the IgG light chain (23 kDa) when utilizing immunoprecipitation techniques to confirm acetylation. Future experiments will be to confirm HDAC8 acetylation substrates. All four targets have been shown to be overexpressed in numerous cancers and promote tumor progression [26–29].

**Table 1:** Summary of 2D comparison and MS analysis of S462 treated with DMSO and PCI3.

Spot^1^	Protein	Abv	Function	M.W.	Diff.^2^
1	Peroxiredoxin 6	PRDX6	Redox regulation	28,675	4.7
2	Parkinson protein 7	PARK7/DJ-1	Oxidative stress	24,592	3.2
3	Phosphoglycerate mutase 1	PGAM1	Glycolysis	29,639	3.5
4	High mobility group protein B1	HMGB1	DNA repair	25,798	3.0

^1^ Corresponding gel location/spot in Fig 4

^2^ PCI3 vs DMSO difference. Differences are calculated from spot percentages.

Some acetylation spots are lost after PCI3 treatment vs DMSO treatment (Fig 4). Kramer et al., demonstrated that the utility of pan-HDAC inhibitors and HDAC6 inhibitors can hyperacetylate α-tubulin and deplete acetyl-CoA resulting in the loss of acetylation sites on acetylated proteins [30]. Loss of acetylated spots after PCI3 treatment may be due to a similar mechanism involving acetyl-CoA as described by Kramer and colleagues.

## Discussion

Our data demonstrates a potential tumorigenic role of HDAC8 in MPNST. Similar to T-cell-derived leukemia [12] and neuroblastoma cells [13], [15], our human and murine-derived MPNST cells exhibited “sensitivity” to HDAC8 inhibition. Interestingly, neuroblastoma and MPNST arise from the neural crest and thus may play an underlying role in their similar responses to HDAC8 inhibition as compared to other tumor types.

A unique observation in our study was HDAC8 inhibition-induced S-phase arrest in MPNST regardless of NF1 status or species of cell line origin. S-phase cell cycle arrest occurred using either HDAC8 inhibitor (PCI3/PCI4), whereas pan-HDAC inhibitor PCI2 induced G2-cell cycle arrest in sporadic human MPNST cells as well as human and murine-derived NF1-associated MPNST cells.

We are currently exploring the role of HDAC8 in S-phase arrest in MPNST. It is encouraging that Deardorff et al. has already demonstrated SMC3 (structural maintenance of chromosomes 3) as a deacetylation substrate of HDAC8 in HeLa cells [31]. The role of SMC3 in MPNST is yet to be determined.

SMC3 is a member of the SMC family of proteins [32], [33] and is vital in the protein cohesin complex which holds sister chromatids together during mitosis, enabling proper separation of the chromosome [33]. Zhang and colleagues demonstrated that acetylation of SMC3 occurs and plays a critical role during S-phase sister chromatid cohesion in yeast and human biological systems [33]. We demonstrated potential S-phase arrest after PCI3/4 treatment, whereas Deardorff and colleagues demonstrated unchanged cell cycle progression in HeLa cells after HDAC8 inhibition using PCI-34051; a difference possibly due to cell type and content. Further study of HDAC8-SMC3 interaction may augment current understanding regarding the role of HDAC8 in cell cycle control. If HDAC8 inhibition results in S-phase cell cycle arrest in MPNST cells, it may be worthwhile to consider combining HDAC8 inhibitors with antimetabolitic compounds (e.g. 5-fluorouracil, cytarabine, gemcitabine), which exert their cytotoxic effects during S-phase [34–36].

Currently there are few identified HDAC8 acetylation targets [37]. Recently, Karolczak-Bayatti and colleagues showed that HDAC8 can interact with Hsp20 to affect its acetylation [38]. They used a HDAC8 inhibitor that augmented Hsp20 acetylation with no increase of histone acetylation or discernible global gene expression changes. Similar to this study, our investigations also did not identify histone acetylation or discernible gene changes in MPNST after HDAC8 inhibition. Among the potential HDAC8 acetylation substrates in S462 cells, PRDX6, PGAM1, and HMGB1 have been shown to be acetylated [39–41]; PARK7/DJ-1 acetylation has yet to be reported.
